# Blunt abdominal trauma to a pregnant woman resulting in a child with hemiplegic spastic cerebral palsy and permanent eye damage

**DOI:** 10.1186/1756-0500-6-517

**Published:** 2013-12-06

**Authors:** Elmuntasir Taha, Khalid Nasralla, AbdulRahman Khalid, AbdelAziem A Ali

**Affiliations:** 1 National Ribat University, Khartoum, Sudan; 2 Department of Obstetrics and Gynecology, Faculty of Medicine, Kassala University, P.O. Box 496, Kassala, Sudan

**Keywords:** Trauma, Pregnancy, Eye, Cerebral palsy, Sudan

## Abstract

**Background:**

In today's life trauma is a common and important complication of pregnancy and remains one of the major contributors to maternal and fetal morbidity and mortality.

**Case presentation:**

The authors reported a case of 4 years old child with hemiplegic spastic cerebral palsy and permanent left eye damage due to antenatal trauma. He was an off spring to a 33 years old woman gravida 6 para 5 from western Sudan, who sustained a domestic blunt abdominal trauma during her routine daily activities. The abdominal trauma occurred during the third trimester at 36^th^ week gestation of the pregnancy when the mother hit herself by the woody part of an axe non intentionally.

**Conclusions:**

The findings from this case conclude that relatively minor trauma can have significant adverse effects on the fetus and can be devastating.

## Background

In today's life trauma is a common and important complication of pregnancy and remains one of the major contributors to maternal and fetal morbidity and mortality [1,2]. Even minor maternal trauma can lead to serious complications include maternal injury, death, shock, internal hemorrhage, intrauterine fetal demise, direct fetal injury, abruptio placentae, and uterine rupture [1-4]. The leading causes of obstetric trauma are motor vehicle accidents, falls, assaults, and gunshots, and ensuing injuries are classified as blunt abdominal trauma, pelvic fractures, or penetrating trauma [1-6]. The causes are different with different life styles and different socio-economic and cultural background. We aimed in this case report to highlight the effect of blunt abdominal trauma on the obstetric outcome after we obtained a written consent from the patient for publication of this case report and any accompanied images.

## Case presentation

**Figure 1:**
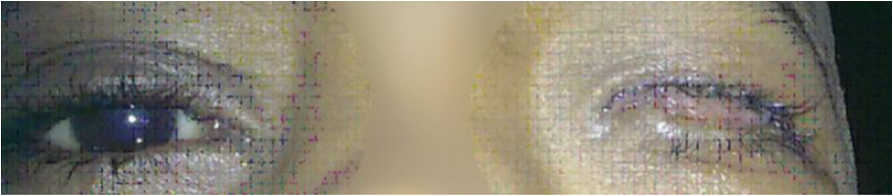
Showing the left eye damage (a child of 4 years old).

**Figure 2:**
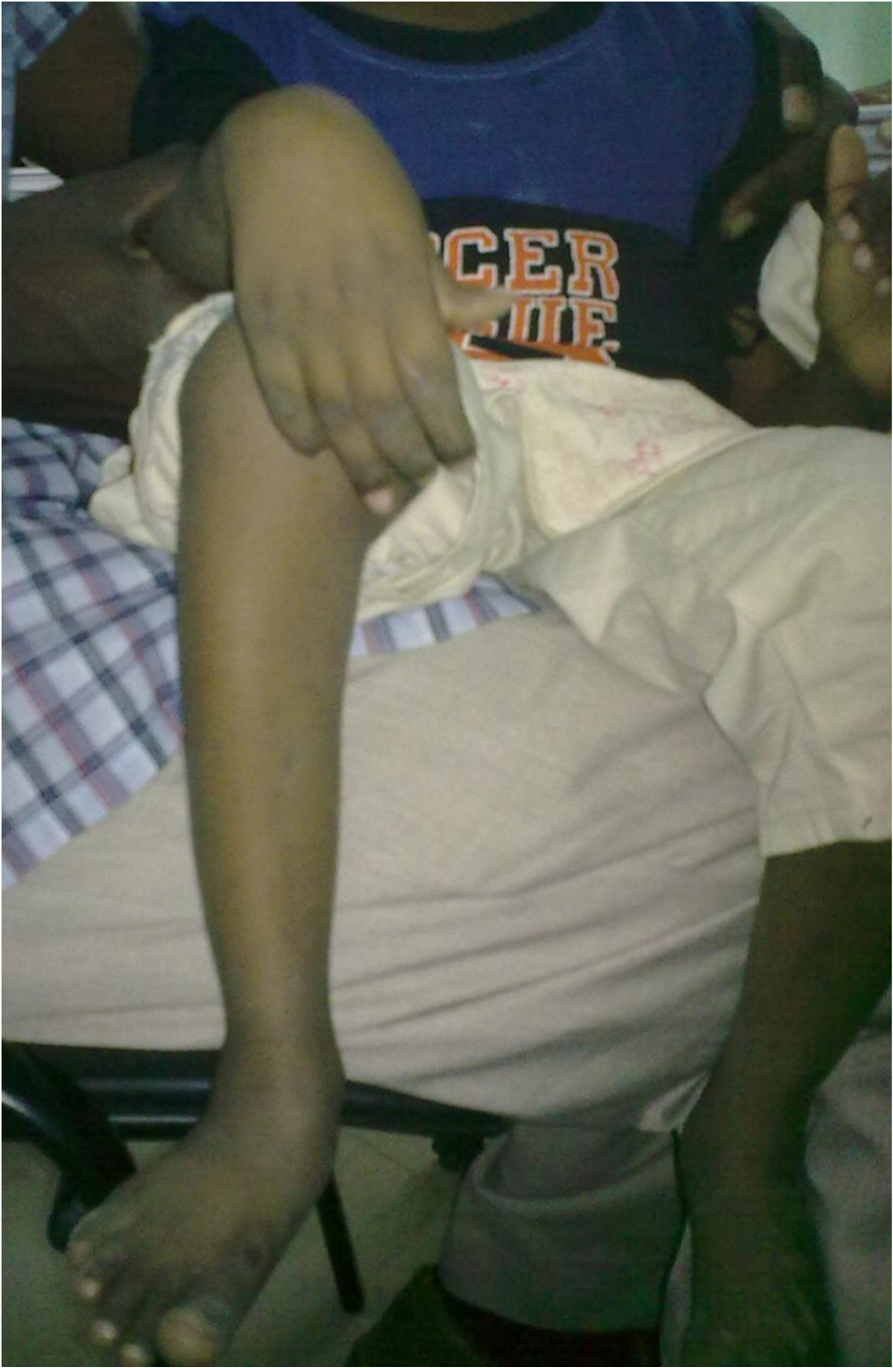
Showing the right sided hemiplegia (a child of 4 years old).

We present a case of 4 years old child with hemiplegic spastic cerebral palsy and permanent left eye damage due to antenatal trauma. He was an off spring to a 33 years old woman gravida 6 para 5, from western Sudan without consanguinity between his parents. The mother sustained a domestic blunt abdominal trauma during her routine daily activities in the year 2009. The abdominal trauma occurred during the third trimester at 36^th^ week gestation, of the pregnancy when the mother hit herself by the woody part of an axe non intentionally. Her pregnancy was uneventful and she had no any significant past medical or obstetrical history. At the time of trauma there was an abnormal fetal movement. Abdominal examination at that time was unremarkable except for a bruise at the trauma site as stated by the referring doctor. Shortly she experienced abdominal pain and vaginal bleeding which necessitated 2 units of blood transfusion in the nearby rural hospital. Four hours later the patient developed uterine contraction which progressed steadily and resulted in vaginal delivery after 10 hours from her admission. She delivered a boy of 2650 gm with an affected left eye and the treating doctor referred her to a tertiary hospital on the second day without recorded data on intrapartum monitoring, neonatal resuscitation and\or Apgar score. At time of presentation the child was irritable with signs of cerebral irritation, his left eye was totally damaged and in addition there was paucity of movement on the right side. The baby was admitted and received the appropriate treatment and then booked for regular follow up to assess the development of his milestone. During the follow up there was global retardation of his milestones. The child ended with right sided hemiplegic spastic cerebral palsy and epilepsy in addition to his eye problem and according to the Growth Motor Function Classification System (GMFCS) the disease was type 4 however the IQ according to the Wechsler Intelligence Scale for children-Revised (WISC-R) was not applicable because the child age < 6 years old. The child was fully investigated, initial CT scan showed brain edema which followed by MRI after discharge which show cerebral atrophy. His eye was managed conservatively and planed for eye inoculation and artificial eye, while multi-disciplinary approached including physiotherapy for his hemiplegia was started (Figures 1 and 2).

## Conclusions

We report a rare case of cerebral palsy as a result of abdominal trauma during the third trimester to highlight the effect of blunt trauma on pregnancy. The effect of trauma on pregnancy depends on the gestational age of the fetus, the type and severity of the trauma, and the extent of disruption of normal uterine and fetal physiology. Trauma occurring during the second and third trimester has different clinical consequences than during the first trimester. First trimester, minor trauma is not threatening to the pregnancy [1-6]. During the second and third trimester, even relatively minor trauma can have significant adverse effects on the fetus. Such adverse effects include placental abruption, preterm labor, uterine rupture, and direct fetal injury. In this case, regular uterine contractions began shortly after the trauma (within 4 hours), progressed steadily and resulted in delivery. Premature rupture of the fetal membranes can also occur, within the first 4 hours of injury and usually result in a premature delivery. Direct fetal injury may occur, resulting in contusions, fractures or fetal death. Uterine rupture can occur and usually result in the loss of the fetus [7]. Eye damage which occurred in our case possibly might be related to the fetal position at time of trauma and the amount of liquor that represent an insulator and could absorb the blunt trauma, while cerebral palsy could be explained by the fetal head injury after the abdominal trauma. The effect of trauma on the pregnant woman and unborn fetus can be devastating. The major causes of maternal injury are blunt trauma, penetrating trauma, burns, falls, and assaults [8]. With the active life-style of today's pregnant women, the effects of trauma have become an important obstetric concern. A protocol was developed to monitor pregnancies complicated by major blunt abdominal trauma in the third trimester, looking specifically for delayed placental and/or fetal problems. In a series of the 84 pregnancies studied, the most serious complication was placental abruption. Although abruption occurred in only two cases, one case was associated with a ruptured uterus and fetal death. There were no cases of delayed abruption or delayed fetal compromise. The most common complication was preterm labor, occurring in 28% of cases when the traumatic insult happened before 37 weeks' gestation. Of these 17 patients, 15 were successfully treated with tocolysis. There were no cases of direct fetal injury or Rhesus-isoimmunization. A revised protocol is recommended for limited outpatient observation with non stress testing and screening ultrasonography to rule out preterm labor and placental abruption and to document fetal well-being [9]. The survival of the fetus after trauma depends on the mother's condition in regard to respiratory passage, oxygenation, and hypovolemia [10]. Thus in conclusion, the findings from this case confirmed that relatively minor trauma can have significant adverse effects on the fetus and can be devastating.

## Consent

Written informed consent was obtained from the mother for publication for this case report and any accompanying images. A copy of the written consent is available for review by the series editor of this journal.

## Competing interests

The authors declare that they have no competing interest.

## Authors’ contribution

TE reported the case, participated in drafting of the manuscript and managed the case. NK and KA participated in drafting of the manuscript and literature review, AAA coordinated the case report and participated in the drafting of the manuscript.
